# Improved Calculations of Heavy Metal Toxicity Coefficients for Evaluating Potential Ecological Risk in Sediments Based on Seven Major Chinese Water Systems

**DOI:** 10.3390/toxics11080650

**Published:** 2023-07-27

**Authors:** Yu Cao, Ruimin Wang, Yanyan Liu, Yongjie Li, Lifen Jia, Qingxiang Yang, Xiangpeng Zeng, Xinlei Li, Qiang Wang, Ruifei Wang, Luqman Riaz

**Affiliations:** 1 College of Life Sciences, Henan Normal University, Xinxiang 453007, China; 2 Henan International Joint Laboratory of Agricultural Microbial Ecology and Technology, Henan Normal University, Xinxiang 453007, China; 3 Department of Environmental Sciences, Kohsar University Murree, Murree 47150, Punjab, Pakistan

**Keywords:** heavy metal pollution, potential ecological risk assessment, toxicity coefficient, release effect

## Abstract

Several methods have been used to assess heavy metal contamination in sediments. However, an assessment that considers both composite heavy metal speciation and concentration is necessary to accurately study ecological risks. This study improved the potential ecological risk index method and calculated the toxicity coefficients of seven heavy metals: Arsenic (As), Cadmium (Cd), Chromium (Cr), Copper (Cu), Nickel (Ni), Lead (Pb), and Zinc (Zn). The newly calculated toxicity coefficients were validated by using previously published heavy metal distribution data of the Henan section of the Yellow River. The calculation procedure is based on the principle that the abundance of heavy metals in the environment and their bioavailable forms affect the toxicity of heavy metals. The toxicity coefficients for the seven heavy metals were calculated as follows: As = 10, Cd = 20, Cr = 5, Cu = 2, Ni = 5, Pb = 5, Zn = 1. Ecological risk assessment of the Henan section of the Yellow River using the improved toxicity coefficients revealed that the ecological risk of Cd and total heavy metals is higher than previous calculations, reaching the strength and moderate risk levels, respectively. The improved potential ecological risk index method is more sensitive to heavy metal pollution and thus provides a better indication of ecological risk. This is a necessary improvement to provide more accurate pollution assessments.

## 1. Introduction

Heavy metals are non-biodegradable elements that have the ability to persist in the natural environment. Their presence has caused significant environmental pollution problems, making heavy metal pollution one of the most serious challenges faced by humankind. In fact, over the course of the past half-century, the global release of heavy metals into the environment has surpassed a staggering 3,000,000 tons [1]. Given the detrimental impact heavy metal pollution can have on ecosystems and human health, it becomes crucial to effectively evaluate and assess this form of pollution. Such evaluation plays a vital role in controlling the emissions of heavy metals and safeguarding the integrity of the ecological environment.

Scholars from various domestic and international backgrounds have proposed numerous evaluation methods from different perspectives. Scientists from countries such as Germany, the United Kingdom, the United States, and Sweden have introduced several assessment approaches for heavy metal pollution from a sedimentological standpoint. Commonly used evaluation methods for heavy metal pollution include the Nemerow Pollution Index, Geological Accumulation Index, Risk Assessment Codes, and Potential Ecological Risk Index [2,3,4]. These evaluation methods have different focuses. For example, the Nemerow pollution index focuses on the evaluation of heavy metal concentrations. The geo-accumulation index focuses on the relationship between total heavy metal concentrations and background values. The risk assessment code focuses on the evaluation of heavy metal speciation. Lastly, the potential ecological risk index assesses the comprehensive impact of heavy metals on the ecological environment.

The Potential Ecological Risk Index (*RI*) proposed by a leading Swedish geochemist named [5], reflects not only the individual effects of various pollutants in a particular environment, but also the combined effects of multiple pollutants, and classifies the potential hazard level in a quantitative way. It is currently one of the most commonly used methods for evaluating the extent of heavy metal pollution. One of the main focuses of this approach is to determine the toxicity coefficients of heavy metals. The study by Chen Jingsheng and Xu Zhengqi recalculated and supplemented the toxicity coefficients of heavy metals [6,7]. However, no studies have considered the bioavailability of heavy metals in the *RI* method.

The main objective of this study was to recalculate toxicity coefficients for Cu, Cr, Pb, Ni, As, Zn, and Cd based on Hakanson’s calculation principles using recently published data about heavy metal speciation and element abundance values in the sediments of seven major water systems in China. The updated toxicity coefficients were used to improve the potential risk evaluation index method. The results of this study provide a computational basis for assessing heavy metal contamination in sediments.

## 2. Materials and Methods

### 2.1. Influencing Factors of Potential Ecological Risk Index

According to Håkanson [5], the potential ecological risk index is based on the following four preconditions:

(1) Content condition: the concentration of metals in the surface sediment. The *RI* value should increase with the increase of surface metal contamination.
(2) Quantitative conditions: the number of metal contaminants. The *RI* value of sediment contaminated with multiple metals should be higher than that of sediment contaminated with only a few metals.
(3) Toxic conditions: the toxicity levels of metals. Different metals have different toxicity levels and metals with higher toxicity should contribute more to the *RI* value than those with lower toxicity.
(4) Sensitivity conditions: the sensitivity of the water body to metal pollution. Water bodies with high sensitivity to metal pollution should have higher *RI* values than water bodies with low sensitivity.

### 2.2. Calculation of Potential Ecological Risk Index

The potential ecological risk index before improvements (*RI*) can be calculated based on Håkanson [5] calculations following Equation (1):

(1) $$RI=\sum_{i=1}^{m}E_{r}^{i}=\sum_{i=1}^{m}\left(T_{r}^{i}\times \frac{C_{D}^{i}}{C_{B}^{i}}\right)$$

*RI* refers to the potential ecological risk index for composite heavy metals for the water system. $E_{r}^{i}$ refers to the potential ecological risk index for single heavy metal. $T_{r}^{i}$ represents the toxicity coefficients which reflect the coefficient, toxic level, and as well as water sensitivity of the metal element heavy metals. $C_{D}^{i}$ and $C_{B}^{i}$ are the heavy metal concentrations measured in the sediment samples and soil, respectively.

The improved potential ecological risk index (*RII*) can be calculated following Equation (2):

(2) $$RII=\sum_{i=1}^{m}{EI}_{r}^{i}=\sum_{i=1}^{m}\left({TI}_{r}^{i}\times \frac{C_{D}^{i}}{C_{B}^{i}}\right)$$

*RII* refers to the potential ecological risk index for composite heavy metals. ${EI}_{r}^{i}$ refers to the improved potential ecological risk factor for a single heavy metal. ${TI}_{r}^{i}$ is the improved toxicity coefficients. $C_{D}^{i}$ and $C_{B}^{i}$ are defined the same as in Equation (1). The difference between Equations (1) and (2) is the calculation of the toxicity coefficients, which will be described in detail below.

### 2.3. Calculation of Metal Toxicity Coefficients

Two key concepts must be considered in determining the toxicity coefficients, which should take into account the “abundance principle” and the “release effect” [5]. The “abundance principle” indicates that the potential toxicological effect of a metal should be proportional to its rarity. The higher the abundance of an element in the environment, the lower its toxicity coefficient should be. Based on this principle, this paper ranks the relative abundances of Cu, Cr, Pb, Ni, As, Zn, and Cd by considering the abundances of heavy metals in igneous rocks, sediments, freshwater, terrestrial plants, and terrestrial animals (Table 1).

In the sediments, the abundance of Cd and As is 531.7 and 7.4 times lower than the abundance of Zn, respectively (e.g., calculated as the Zn abundance of 67 divided by the Cd abundance of 0.126, this provides a relative abundance of 531.7). The relative abundance of these metals was subsequently calculated based on the highest abundance of each metal in each column divided by the concentration of each individual metal element. The relative abundances of the seven metals in each environmental material (rocks, freshwater, terrestrial plants, terrestrial animals, and sediments) required for the calculation of the abundance index are listed in Table 2.

To prevent inappropriate weights from impacting the total abundance, the highest value in each row was omitted to obtain the sum of the remaining four categories, before being divided by 4 to obtain the average abundance index of heavy metals. This abundance index calculation ranks these heavy metals in the following order: Cd > As > Cr > Pb > Ni > Cd > Cu > Zn.

The abundance of metal elements is related to the toxicity coefficients of the elements but is not directly equivalent to the toxicity coefficients, and the “release effect” of the metallic element needs to be considered. The *RI* calculation used the ratio of metal content in water and metal content in preindustrial sediments to represent the “release effect” of metal elements [5].

In this paper, data from seven major water systems in China (the Yangtze River, Yellow River, Huaihe River, Haihe River, Liaohe River, Songhua River, and Zhujiang River) were compiled for analysis. Three sites from each water system were selected to obtain the speciation distribution of heavy metals and to calculate the average bioavailable heavy metal ratios of Chinese sediment heavy metals (Table 3). This average ratio was substituted for the “release effect” of metal elements in *RI*, a consideration mainly because different heavy metal speciation can produce different environmental effects, which directly affect the toxicity and transport of heavy metals [9]. The order of the release coefficients of the seven heavy metals in the sediment calculated by this method is: Cd > Pb > Zn > Ni > Cu > As > Cr. The average abundance index of each heavy metal was multiplied by the release coefficients to obtain the ${TI}_{r}^{i}$ values (Table 4).

The calculated ${TI}_{r}^{i}$ integrates the abundance and bioavailability of heavy metals in the environment. This reflects both the “abundance principle” and the “release effect”, where higher abundance and bioavailability are associated with lower toxicity. In accordance with Håkanson’s method, the ${TI}_{r}^{i}$ values were treated as follows for regularization and convenience in practice. First, the ${TI}_{r}^{i}$ value of each element was divided by 0.29 (the corrected abundance value of zinc, which is the smallest among the seven elements) to obtain the following values: As = 67.53, Cd = 494.02, Cr = 37.32, Cu = 2.22, Ni = 8.82, Pb = 16.27, Zn = 1. Next, we calculate the square-root of these values: As = 8.22, Cd = 22.23, Cr = 6.11, Cu = 1.49, Ni = 2.97, Pb = 4.03, Zn = 1. Finally, the squared value is normalized to provide the updated toxicity coefficient values: As = 10, Cd = 20, Cr = 5, Cu = 2, Ni = 5, Pb = 5, and Zn = 1.

There is a difference between this paper and the evaluation method proposed by Håkanson [5]. Therefore, we adjusted the ${EI}_{r}^{i}$. The original $E_{r}^{i}$ defined “mild risk” for the highest toxicity coefficient of evaluation metals (Mercury = 40), and gradually increased the grade by multiples of 40 for each risk category [5]. The highest toxicity coefficient in this study was 20 (Cd = 20), thus in the same way, the “mild risk” grade was defined as a value less than 20, before the grade is gradually upgraded. In Håkanson’s study, the sum of the toxicity coefficients of pollutants was 133, and the initial value of the composite heavy metal pollution standard was 150. The sum of the seven heavy metals toxicity coefficients in this paper is 44, so the initial value of the pollution standard for composite heavy metals was set as follows: *RII* = 150 × (47/133) ≈ 53.01 (rounded to the integer 50). The adjusted classification of heavy metal pollution is shown in Table 5.

## 3. Results

### 3.1. Improved Heavy Metal Toxicity Coefficients

After taking into account the “abundance principle” and the “release effect”, we have determined the improved heavy metal toxicity coefficients as As = 10, Cd = 20, Cr = 5, Cu = 2, Ni = 5, Pb = 5, and Zn = 1.

### 3.2. Pollution Characteristics of Heavy Metals in the Henan Section of the Yellow River

**Figure 1:**
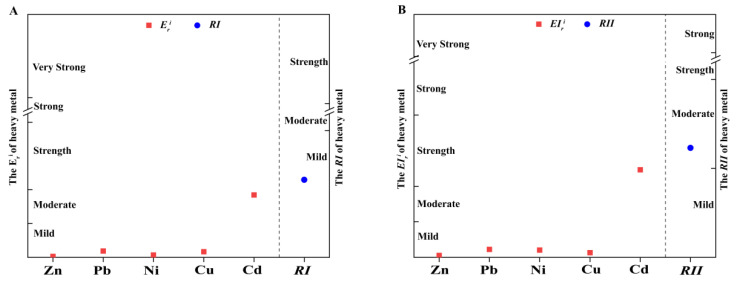
Potential ecological risk assessment of the Henan section of the Yellow River. (**A**) Potential ecological risk index reported in the literature. (**B**) Potential ecological risk index calculated using improved toxicity coefficients. *RI* and *RII* refer to the potential ecological risk index of composite heavy metals before and after improvement, respectively. $E_{r}^{i}$ and ${EI}_{r}^{i}$ refer to potential ecological risk factors for individual heavy metals before and after improvement, respectively. The red color indicates the potential ecological risk for each metal, and the blue color indicates the potential ecological risk index for composite heavy metals.

The *RI* of heavy metals in the Henan section of the Yellow River has been revealed in the literature using the potential ecological risk index method [16]. In this study, we used the *RII* values to compare with previously published *RI* values (Figure 1). Both methods indicated that Cd was the main contributor to the ecological risk in the Henan section of the Yellow River, and the other metals were rated at a lower risk level than Cd. However, the potential ecological risk evaluation results of the two methods were not consistent, since the improved calculation suggests a higher risk level for Cd and total heavy metals. The ${EI}_{r}^{i}$ of Cd and *RII* are rated at the strength and moderate risk levels, respectively, which are both one level higher than the $E_{r}^{i}$ of Cd and *RI* calculated in Håkanson [5].

## 4. Discussion

### 4.1. Improved Heavy Metal Toxicity Coefficients

The deposition of sediment serves as a significant source and storage mechanism for heavy metals in surface water environments, playing a crucial role in their transport and accumulation [31]. Heavy metal pollution in sediments is not only influenced by natural factors but also by anthropogenic sources such as fossil fuel combustion, metal smelting, coal and electroplating industries, domestic sewage, and aquaculture [32,33]. Wang investigated the metal elements in the surface sediments of Chaohu Lake in eastern China and found that Pb, Zn, and Cd had the highest concentrations in the samples [34]. Zhang studied the risk of heavy metals in shallow lake sediments in eastern China and identified Cd as having a high risk [35]. Additionally, it has been reported in the literature that aquaculture areas are subjected to more severe pollution of arsenic, nickel, copper, and zinc compared to non-aquaculture areas [36]. Kang discovered that Cr and Pb were the primary contributors to health risks in the surface sediments of the Hai River and its tributaries in Tianjin, with the carcinogenic risk associated with Cr being two orders of magnitude higher than that of Cd [12]. Considering the extensive literature reporting on the metals As, Cd, Cr, Cu, Ni, Pb, and Zn, this study selected these seven heavy metals as the research focus.

After comparing our toxicity ranking sequence with that reported in the literature, we found that the overall results were mostly consistent. However, the order was different for Cu and Cr. In the literature, Cd, As, and Cu were reported to have the highest toxicity ranking [5], while in this study, Cd, As, and Cr had the highest toxicity ranking. This discrepancy can be attributed to the difference in the release coefficients. Specifically, in the study conducted by Hakanson [5], the ratio of metal content in water to that in industrial sediment was used to represent the “release effect” of metal elements. It was found that the release effect of Cu was 100 times greater than that of Cr [5]. However, in this study, we argue that metal bioavailability is a better indicator of the release effect of metal elements. There have been reports indicating that the speciation of heavy metal elements directly relates to their leaching rate, migration in the environment, and bioavailability [37]. Heavy metals can form various chemical species when combined with substances in the soil, and their speciation not only determines their migration capability but also has a direct impact on their accumulation capacity and biological toxicity [38]. Through the analysis of metal speciation in the sediments of seven major river basins in China, we found that the release coefficient of Cu was only 2.2 times that of Cr. Furthermore, considering that the abundance index of Cr in the environment is much higher than that of Cu (Cr = 123.1, Cu = 3.3), this explains why the ecological risk of Cr is higher than that of Cu in this study. Previous studies have also reported that Cr is more toxic to organisms than Cu [39,40].

### 4.2. Pollution Characteristics of Heavy Metals in the Henan Section of the Yellow River

In a previous study conducted by Zhang, potential health risks associated with heavy metals (HMs) in the Yellow River, Henan section, were assessed using the potential ecological risk method [16]. Their findings revealed that Cd was identified as the major contributor to potential ecological risks, with its concentration at a moderate level. However, the overall concentration of heavy metals remained significantly below the threshold for moderate risk. In this current study, we aimed to reassess the risks posed by heavy metals by employing new toxicity coefficients. Interestingly, our results differed from the original study. While Cd still emerged as the major contributor to potential ecological risks, the use of improved toxicity coefficients led to an increase in both the risks associated with Cd and total heavy metals. These new assessments indicated that Cd levels now reached levels of strength, while total heavy metals approached moderate risk levels. This highlights the importance of employing improved toxicity coefficients in regulating Cd and heavy metal pollution. The health risks associated with Cd concentrations are particularly alarming for humans. Even low levels of exposure to Cd can have adverse effects on various organs such as the kidneys, liver, lungs, and cardiovascular system, as established by Brdari and Zeng et al. [41,42]. Therefore, it is crucial to address and mitigate cadmium pollution due to its potential threat to human health. By utilizing the improved assessment method for potential ecological risks, it becomes possible to obtain a more accurate evaluation of heavy metal pollution and gain a better understanding of the actual levels of potential ecological risk involved.

### 4.3. The Applicability of the Improved Heavy Metal Toxicity Coefficients

The Yangtze River, Yellow River, Huai River, Hai River, Liao River, Songhua River, and Pearl River are regarded as the seven major river systems in China, playing an imperative role in the country’s economic development and social stability. It is remarkable that even the smallest basin area, the Liao River, encompasses an expansive 229,400 square kilometers. This paper focuses on the assessment of the release effects of heavy metals in these significant river systems in China, relying on data obtained from extensive research. To ensure the applicability of the methodology in assessing the risks associated with heavy metals in large-scale river systems, three references were carefully selected for each individual river system. Nonetheless, it is important to acknowledge certain limitations. The lack of bioavailability data for specific heavy metals in some river systems, coupled with the vast distribution of the seven major river systems, results in notable variations in metal bioavailability across different regions. Consequently, the methodology presented in this paper might not entirely represent small-scale sampling or rivers in other countries, including certain small tributaries. Researchers interested in studying specific tributaries are encouraged to utilize datasets of local heavy metal morphology while recalculating metal release effects using the methodology presented herein, which can result in more appropriate and accurate toxicity coefficients. This approach allows for a comprehensive understanding of the potential risks posed by heavy metals in specific regions, facilitating more targeted and effective mitigation strategies.

## 5. Conclusions

In this study, the potential ecological risk index proposed by Håkanson was improved to provide updated toxicity coefficients, allowing for the recalculation of evaluation criteria for heavy metals in Chinese sediments. The main findings are as follows:

1. The toxicity coefficients of the seven heavy metals examined in this paper rank as follows: Cd > As > Cr = Ni = Pb > Cu > Zn, with toxicity coefficients of 20, 10, 5, 5, 5, 2, 1, respectively. These updated values provide a basis for calculating the degree of heavy metal pollution in sediments.
2. Cd, As, and Cr are more toxic, and their emissions should be strictly controlled during the production, manufacturing, and disposal of items containing these metals.
3. Compared to the original *RI* formulation, the improved *RII* calculation is more sensitive to heavy metal pollution and thus provides a better indication of ecological risk. This is a necessary improvement to provide more accurate pollution assessments.

## Figures and Tables

**Table 1:** The abundance of metal elements (in ppm) in different physical matter.

Element	Igneous Rocks	Fresh Water	Land Plants	Land Animals	Sediments
As	1.8	0.0004	0.2	0.2	9
Cd	0.2	0.00031	0.6	0.5	0.126
Cr	100	0.00018	0.23	0.075	54
Cu	55	0.01	14	2.4	20
Ni	75	0.01	3	0.8	23
Pb	12.5	0.005	2.7	2	23
Zn	70	0.01	100	160	67

Note: Mass values for heavy metal abundance in igneous rocks, fresh water, land plants and animals are from Håkanson [5]. Sediment values are from Shi [8].

**Table 2:** The relative abundance of elements in different matter types.

Element	Igneous Rocks	Fresh Water	Land Plants	Land Animals	Sediments	$\sum_{i=1}^{4}$	Abundance Index
As	55.6	25.0	500.0	800.0	7.4	588.0	147.0
Cd	500.0	32.3	166.7	320.0	531.7	1018.9	254.7
Cr	1.0	55.6	434.8	2133.3	1.2	492.6	123.1
Cu	1.8	1.0	7.1	66.7	3.4	13.3	3.3
Ni	1.3	1.0	33.3	200.0	2.9	38.6	9.6
Pb	8.0	2.0	37.0	80.0	2.9	50.0	12.5
Zn	1.4	1.0	1.0	1.0	1.0	4.0	1.0

Note: “$\sum_{i=1}^{4}$ ” refers to the sum of four of the five categories of igneous rocks, freshwater, land plants, land animals, and sediments with the maximum value removed.

**Table 3:** Proportion of bioavailable heavy metals in the sediments of Chinese aquatic systems.

Region	Proportion of Bioavailable Heavy Metals						
	As	Cd	Cr	Cu	Ni	Pb	Zn
Haihe river	—	21.30%	3.10%	3.80%	3.20%	5.40%	11.17%
	—	—	—	27.10%	5.10%	8.70%	10.80%
	—	30%	9%	7%	11.50%	11.50%	23%
Huaihe river	—	90%	42%	75%	48%	95%	15%
	—	53.10%	0.50%	4.70%	—	2.80%	16.50%
	—	27.55%	1.80%	6.40%	—	0.30%	55.60%
Yellow River	—	32.00%	—	31.00%	14.50%	48.00%	13.00%
	—	11.80%	10.10%	17.80%	15.70%	10.10%	—
	—	—	3.00%	7.50%	7.50%	21.50%	7.50%
Liao river	10.16%	27.42%	0.32%	5.48%	12.64%	4.93%	8.84%
	—	23.00%	—	17.00%	—	0.30%	11.00%
	—	—	—	7.29%	—	22.19%	16.86%
Songhua	—	—	4.20%	2.37%	59.36%	83.24%	46.33%
	40.00%	60.00%	—	40.00%	40.00%	85.10%	40.00%
	—	98.20%	—	53.00%	—	83.70%	76.30%
Changjiang river	—	—	21.60%	41.15%	34.05%	58.40%	—
	5.00%	42.00%	2.00%	10.20%	6.00%	14.00%	5.00%
	1.00%	29.00%	5.00%	11.00%	14.00%	13.00%	14.00%
Zhujiang water	—	56.60%	13.70%	4.50%	16.70%	49.00%	59.90%
	—	94.50%	49.50%	63.00%	68.00%	26.00%	81.00%
	—	—	24.00%	45.50%	35.00%	61.00%	42.00%
Median	7.58%	32.00%	5.00%	11.00%	15.10%	21.50%	16.50%

Note: The data required in the table are all derived from previous research [10,11,12,13,14,15,16,17,18,19,20,21,22,23,24,25,26,27,28,29,30]. “—” represents that relevant data could not be found in the literature.

**Table 4:** Improved toxicity coefficients of heavy metal elements in Chinese water systems.

Element	Release Factor	Abundance Index	Toxicity Coefficient $\left({TI}_{r}^{i}\right)$
As	0.0758	147.00	11.1426
Cd	0.32	254.73	81.5136
Cr	0.05	123.14	6.157
Cu	0.11	3.33	0.3663
Ni	0.151	9.64	1.45564
Pb	0.215	12.49	2.68535
Zn	0.165	1.00	0.165

**Table 5:** The adjusted criteria for the classification of heavy metal pollution in sediments.

${EI}_{r}^{i}$	Pollution Degree	*RII*	Pollution Degree
<20	Mild	<50	Mild
20–40	Moderate	50–100	Moderate
41–80	Strength	101–200	Strength
81–160	Strong	>200	Strong
161–320	Very Strong		

Notes: “${EI}_{r}^{i}$” refers to the potential ecological risk factor for a single heavy metal. “*RII*” refers to the potential ecological risk index for composite heavy metals.

## Data Availability

No new data were created or analyzed in this study. Data sharing is not applicable to this article.
